# Neural Correlates of Emotion Regulation and Associations With Disordered Eating During Preadolescence

**DOI:** 10.1002/dev.70009

**Published:** 2024-12-08

**Authors:** Kai S. Thomas, Catherine R. G. Jones, Marc O. Williams, Ross E. Vanderwert

**Affiliations:** ^1^ School of Psychology Cardiff University Cardiff UK; ^2^ Cardiff University Brain Research Imaging Centre, School of Psychology Cardiff University Cardiff UK

**Keywords:** disordered eating, emotion recognition, emotion regulation, internalizing symptoms, preadolescence

## Abstract

Difficulties with emotion regulation have been documented in individuals with eating and internalizing disorders. However, there is limited research examining the cognitive processes underlying these difficulties. Using a dimensional approach, the current study examined the link between the behavioral and neural correlates of response inhibition, disordered eating, and internalizing symptoms in a community sample of preadolescents. A total of 50 children (*M* age = 10.9 years; 58% male) completed an emotion Go/No‐Go task, while ERP components were recorded, as well as self‐report measures of disordered eating and internalizing symptoms. In addition, children completed an emotion recognition task to establish whether there were fundamental differences in emotion recognition across high and low levels of disordered eating and internalizing symptoms. Increased disordered eating was associated with increased mean P3‐NoGo amplitudes when inhibiting responses to happy facial expressions, as well as poorer recognition of happy faces. These associations were not found for internalizing symptoms. Our findings suggest an early disruption in response inhibition, specifically for happy emotional expressions, may be relevant to the development of disordered eating behaviors in preadolescence.

## 1 Introduction

Eating disorders (EDs) are mental health conditions that present as persistent disturbances in eating behaviors and eating‐related cognitions (American Psychiatric Association 2013). EDs are characterized by disordered eating (DE) behaviors, including dietary restriction, binge eating, purging, and preoccupying cognitions around eating, weight, and body shape. These behavior patterns also commonly occur in the general population, although in a less severe and infrequent form than clinical populations (Naor‐Ziv and Glicksohn 2016). Subclinical DE behaviors are found to emerge in preadolescence (Hilbert et al. 2013; Neumark‐Sztainer et al. 2011) and are a predictor of developing a diagnosable ED in adolescence (Evans et al. 2017; Herle et al. 2020; Kotler et al. 2001; Tanofsky‐Kraff et al. 2011). Identifying potential risk factors during the early emergence of DE is therefore critical for informing better treatment and prevention of chronic and severe full syndrome EDs.

Emotion regulation is often defined as a complex combination of processes that govern the expression, timing, and intensity of emotional experiences to serve a larger goal (Gross and Thompson 2007). One such process is response inhibition, which forms part of a collection of inhibitory control processes and is defined as the ability to withhold a prepotent incorrect response to perform a correct response and maintain goal performance (Davidson et al. 2006). Evidence suggests response inhibition plays a crucial role in emotion regulation by enabling individuals to override their default emotional expressions and maintain goal‐directed behavior (Pruessner et al. 2020), such as inhibiting a negative emotional response in a socially inappropriate context. Emotion regulation is a key factor involved in the development and maintenance of EDs (Harrison et al. 2010; Henderson et al. 2021; Lavender et al. 2015). Studies exploring emotion–regulation deficits in individuals with a diagnosed ED have reported significantly higher levels of experienced emotion intensity, less acceptance and awareness of emotions, limited expression of emotions, increased rumination about these emotions, as well as more self‐reported emotion regulation problems when compared to healthy controls (Boscoe, Stanbury, and Harrison 2021; Fox et al. 2013; Svaldi et al. 2012). These difficulties are also present in children and early adolescents with DE (McLaughlin et al. 2011; Sim and Zeman 2006). However, research examining the behavioral and neural correlates underlying these processes, such as response inhibition, is limited.

Internalizing symptoms, such as anxiety and depression, are commonly found to co‐occur with EDs and DE across the lifespan (Evans et al. 2017; Hudson et al. 2007; Thomas et al. 2021; Touchette et al. 2011; Ulfvebrand et al. 2015) and share in a similar emotion regulation phenotype (Hallion, Tolin, and Diefenbach 2019; MacNamara et al. 2017; Telzer et al. 2008). There is now a substantial body of research demonstrating the important role of response inhibition impairments across mental health conditions characterized by emotion regulation difficulties, including EDs and internalizing disorders (Bartholdy et al. 2016; Grillon et al. 2017; Li et al. 2021; Thomas et al. 2024; M. Wu et al. 2013). However, previous research in this area has focussed on EDs and internalizing disorders in isolation; so it is not clear whether internalizing symptoms may be driving any response inhibition effects observed in people with EDs, especially in emotional contexts.

Go/NoGo tasks are commonly used to measure response inhibition abilities (Hare et al. 2005). They require the participant to perform speeded responses on Go trials, such as a button press, and withhold a response on NoGo trials. Emotional Go/NoGo tasks are adapted to use affective stimuli, such as emotional facial expressions or other emotionally valent stimuli. Recent research using an emotional Go/NoGo task with a non‐clinical sample found the link between difficulties in emotion regulation and DE to be stronger for individuals with greater response inhibition difficulties, but only when presented with pleasant stimuli (Ramos et al. 2024). Emotional Go/NoGo tasks have also been used alongside electroencephalography (EEG) in children and adolescents with internalizing symptomatology to capture behavioral and neural markers of response inhibition during the presentation of emotional facial expressions (Hum, Manassis, and Lewis 2013a, 2013b; Lewis et al. 2008; Lewis et al. 2006; Lewis, Todd, and Honsberger 2007; W. Zhang et al. 2016).

The neural correlates of response inhibition include two stimulus‐locked event‐related potentials (ERPs) generated in the EEG, the N2 and P3, located over frontocentral sites. The N2 component is a negative deflection in amplitude around 200 ms post‐stimulus that is greater in NoGo trials compared to Go trials and reflects monitoring conflict between competing responses (e.g., Albert et al. 2013; Donkers and Van Boxtel 2004; Hong et al. 2017). The P3 is a positive deflection following the N2 that is greater in NoGo trials compared to Go trials, associated with inhibitory processing and evaluation of the conflict stimulus (Bruin, Wijers, and van Staveren 2001; Hong et al. 2017; Wessel 2018). Using emotional Go/NoGo tasks in children and adolescents with internalizing symptomatology, studies report enhanced neural correlates of response inhibition during presentations of *both* positive and negative stimuli compared to healthy controls (Hum, Manassis, and Lewis 2013a; Lewis et al. 2008). For depression, some studies report reduced N2 and P3 amplitudes for positive emotions (Camfield et al. 2018), but others report enhanced P3 amplitudes (W. Zhang et al. 2016). Interestingly, these atypical neural markers are not accompanied by behavioral task impairments in children with anxiety (Hum, Manassis, and Lewis 2013a, 2013b; Lewis et al. 2008; Waters and Valvoi 2009) or depression (Grunewald et al. 2015; Trinkl et al. 2015). This may suggest that neural differences are observable before behavioral differences have emerged, highlighting the importance of examining both neural and behavioral components at earlier stages of development.

Although emotional Go/NoGo tasks have not been previously used alongside EEG in studies with people with EDs or DE, attenuated early ERP components to emotional faces have been reported in adolescents with anorexia nervosa compared to healthy controls (Hatch et al. 2010; Sfärlea et al. 2016), indicating dysfunctions in automatic and perceptual processing. There is evidence that individuals with EDs may also present with dysfunctions in later and more complex processing. The P3, for example, is found to be smaller during emotional processing in individuals with anorexia nervosa (Hatch et al. 2010; Pollatos et al. 2008). Importantly, as with findings from anxiety and depression studies, these atypical neural markers are not accompanied by behavioral impairments in individuals with EDs (Hatch et al. 2010; Sfärlea et al. 2016).

In sum, cognitive control processes, such as response inhibition, are thought to be involved in emotion regulation. Behavioral and neural correlates of response inhibition have been previously studied in the context of emotion in children with anxiety and depression. However, to our knowledge, no study yet has examined the behavioral and neural correlates of response inhibition in children with DE during the presentation of emotional facial expressions. The current study aimed to address this gap by investigating associations between DE behaviors, internalizing symptoms, and both behavioral and neural correlates of response inhibition to emotional stimuli in a community sample of preadolescents. Due to the overlap between automatic emotion regulation processes and emotion recognition (Ochsner 2008), as well as the consistent emotion recognition difficulties reported in adults with EDs (Harrison et al. 2009) and both children and adults with internalizing symptoms (Collin et al. 2013; Demenescu et al. 2010; Rappaport et al. 2021; Surcinelli et al. 2006), emotion recognition performance will also be assessed to establish whether there are fundamental differences in emotion recognition that could be driving any of these emotion‐regulation effects.

We hypothesized that increased levels of DE and internalizing symptoms will be positively correlated with neural markers of impaired response inhibition (in the form of less positive P3 amplitudes and more negative N2 amplitudes on NoGo trials). Due to the comparable behavioral performance reported between children with internalizing symptoms, adults with EDs, and healthy controls, we did not expect to find significant associations between behavioral markers of response inhibition and both DE and internalizing symptoms. Due to the high co‐occurrence between internalizing symptoms and DE, this study further aims to examine whether response inhibition difficulties were associated with DE *independently* of internalizing symptoms, or whether internalizing symptoms mediated the relation *between* these processes.

## 2 Methods

### 2.1 Participants

A total of 63 participants (*M* age = 11.0 years, SD = 0.49; 46.0% female) were recruited for this study across two stages. During the Stage 1, 26 children (*M* age = 10.9 years; 53.8% female) were recruited from a previous stage of the project (details provided in Thomas et al. 2024). These children completed the self‐report questionnaires in their school and were invited to participate in the current study at the University between August 2019 and March 2020. Typically, there was a delay of 2–3 months between participants completing the questionnaires in their school and participating in the current study. Stage 2 of recruitment invited 37 children (*M* age = 11.0 years; 40.5% female) to participate in the study through social media advertisements and invitations through a recruitment database. Due to disruptions to testing during the COVID‐19 pandemic, this took place during March–September 2021. Invitation emails and social media advertisements to families across both recruitment stages described the research as an investigation of children's brain activity and how it related to their eating behaviors, thoughts, and feelings. A *t*‐test revealed that the two recruitment groups did not differ significantly in child age (*t*(61) = 1.04, *p* = 0.31) or parent age (*t*(60) = −1.75, *p* = 0.09). There were no significant differences in the number of boys and girls recruited at each recruitment stage (*X* ^2^ (1, *N* = 63) = 1.09, *p* = 0.30), reported socio‐economic status (*U* = 395.5, *z* = −1.25, *p* = 0.21), or ethnicity (*X* ^2^ (3, *N* = 60) = 6.47, *p* = 0.09). Finally, we compared the levels of DE and internalizing symptoms and found significant differences across the two recruitment types (DE: *t*(61) = 2.19, *p* = 0.03; anxiety: *t*(61) = 2.59, *p* = 0.01; depression: *t*(61) = 3.16, *p* = 0.01). For all the three measures, the children recruited pre‐pandemic in Stage 1 reported higher levels of DE, anxiety, and depressive symptoms compared to the children recruited in Stage 2. Analyses were conducted separately within the pre‐ and post‐pandemic groups and the patterns of results were consistent with those reported for the entire sample, unless specified.

We report how we determined our sample size, all data exclusions (if any), all manipulations, and all measures in the study. First, parents confirmed their child did not meet any of the following exclusion criteria: premature birth, significant developmental delays, uncorrected visual difficulties, or significant head trauma leading to neurological abnormalities. Concerning task exclusionary criteria, 10 children were excluded based on low task accuracy on the Go/No‐Go task (accuracy < 50%), resulting in a final sample of 53 participants (*M* age = 10.98; 56.6% male). One participant was excluded from all ERP analyses due to excessive EEG artifacts. The sample size was determined through comparisons with similar ERP studies exploring emotion regulation in preadolescents (Connell et al. 2020; Hum, Manassis, and Lewis 2013a; Liu et al. 2022). A post‐hoc sensitivity analysis was also conducted to determine the effect size that could be detected based on the collected sample size. On the basis of a two‐tailed bivariate correlation with an *α* value of 0.05, power of 0.95, and sample size of 53, the critical *r* = 0.271.

The project received approval from the University School of Psychology Ethics Committee (EC.19.02.12.5566GR5A6). Written informed consent from the parent/guardian and child assent was obtained before the experiment began. As compensation for their time, each child received a gift voucher and a small gift. Table 1 presents the demographics of the final sample once exclusion criteria were employed. Socio‐economic status was determined via postcode matching to the Welsh Index of Multiple Deprivation.

**TABLE 1:** Characteristics of the children in the final sample (*N* = 53).

Demographics	*M* (range)		
Age (years)	10.98 (10.00–11.83)		
Gender (male %)	56.6		
Ethnicity (%)			
White	83.0		
Mixed or multiple ethnic groups	3.8		
Asian or Asian British	1.9		
Other ethnic group	7.5		
Black, African, Caribbean or Black British	0		
Missing data	3.8		
SES (WIMD) quartile (%)			
First (most deprived)	24.5		
Second	18.9		
Third	18.9		
Fourth (least deprived)	37.7		

Descriptive statistics for questionnaire measures	*M* (SD)	Range	Possible Range
ChEAT	58.40 (12.96)	35–100	26–156
RCADS Anxiety	11.19 (7.25)	1–32	0–45
RCADS Depression	7.36 (4.99)	0–20	0–30

Go/No‐Go counterbalance condition			Frequency (%)
Go response = Female; NoGo response = Male			25 (47.2)
Go response = Male; NoGo response = Female			28 (52.8)

Abbreviations: ChEAT, Children's Eating Attitude Test; RCADS, Revised Child Anxiety Depression Scale; SES, Socio‐economic status; WIMD, Welsh Index of Multiple Deprivation.

### 2.2 Self‐Report Questionnaire Measures

#### 2.2.1 Children's Eating Attitude Test

DE behaviors and attitudes were measured using the Children's Eating Attitude Test (ChEAT; Maloney et al. 1989), a 26‐item self‐report modified version of the abbreviated adult Eating Attitudes Test (EAT‐26; Garner and Garfinkel 1979). Using the traditional scoring strategy, the three most symptomatic responses (“often,” “very often,” and “always”) are scored 1–3, and the remaining three responses (“never,” “very rarely,” and “rarely”) were scored as 0. This approach to scoring limits the variability of the data (Anton et al. 2006; Smolak and Levine 1994). Therefore, the current study employed an alternative scoring strategy, which has been previously used in a large community sample of children aged 7–12 years and resulted in greater variability in item scores and a reduction in skewness for the total ChEAT score (Anton et al. 2006). In the alternative scoring procedure, a Likert scale from 1 (*never*) to 6 (*very often*) was used with all items summed to create a total score. The total score could range within 26–156, with higher scores representing more difficulties.

Adjustments to the wording of items were made to enhance comprehension. Item 4 was changed from “I have gone on eating binges where I feel that I might not be able to stop” to “I have started to eat and then felt like I cannot stop” (see Coombs et al. 2011). Items 9 and 26, which refer to “vomit,” were also accompanied by “am/be sick.” Finally, Item 21 was changed from “I give too much time and thought to food” to “I spend too much time thinking about food.” Cronbach's *α* value for all items using the alternative scoring strategy was acceptable (*α* = 0.713), a slight improvement on the traditional scoring method (*α* = 0.710).

#### 2.2.2 Revised Child Anxiety and Depression Scale

The revised Child Anxiety Depression Scale—25‐item version (RCADS‐25; Muris, Meesters, and Schouten 2002) is a brief assessment of anxiety and depression symptoms as defined in *Diagnostic and Statistical Manual of Mental Disorders* (5th edition) (American Psychiatric Association 2013). The anxiety and depression subscales are comprised of 15 and 10 items, respectively. All the 25 items are rated on a 4‐point scale (*never*, *sometimes*, *often*, *always*) and represent the frequency to which these behaviors, thoughts or feelings occur (e.g., “I have trouble sleeping”). Individual responses are scored from 0 (*never*) to 3 (*always*), with scores calculated by summing the item responses for the anxiety and depression subscales separately. This results in anxiety scores ranging 0–45 and depression scores ranging 0–30. Higher scores indicate more severe anxiety and depression symptomatology. The RCADS‐25 is comparable to the full‐length version regarding test–retest reliability (*r*s = 0.78–0.86, *p* < 0.001) and internal consistency (*α* = 0.87–0.95; Brown et al. 2014). Cronbach's *α* values for the current sample were acceptable for both scales (anxiety: *α* = 0.86; depression: *α* = 0.84), as well as the total score (*α* = 0.92).

### 2.3 Behavioral Tasks

#### 2.3.1 Go/No‐Go Task

**FIGURE 1:**
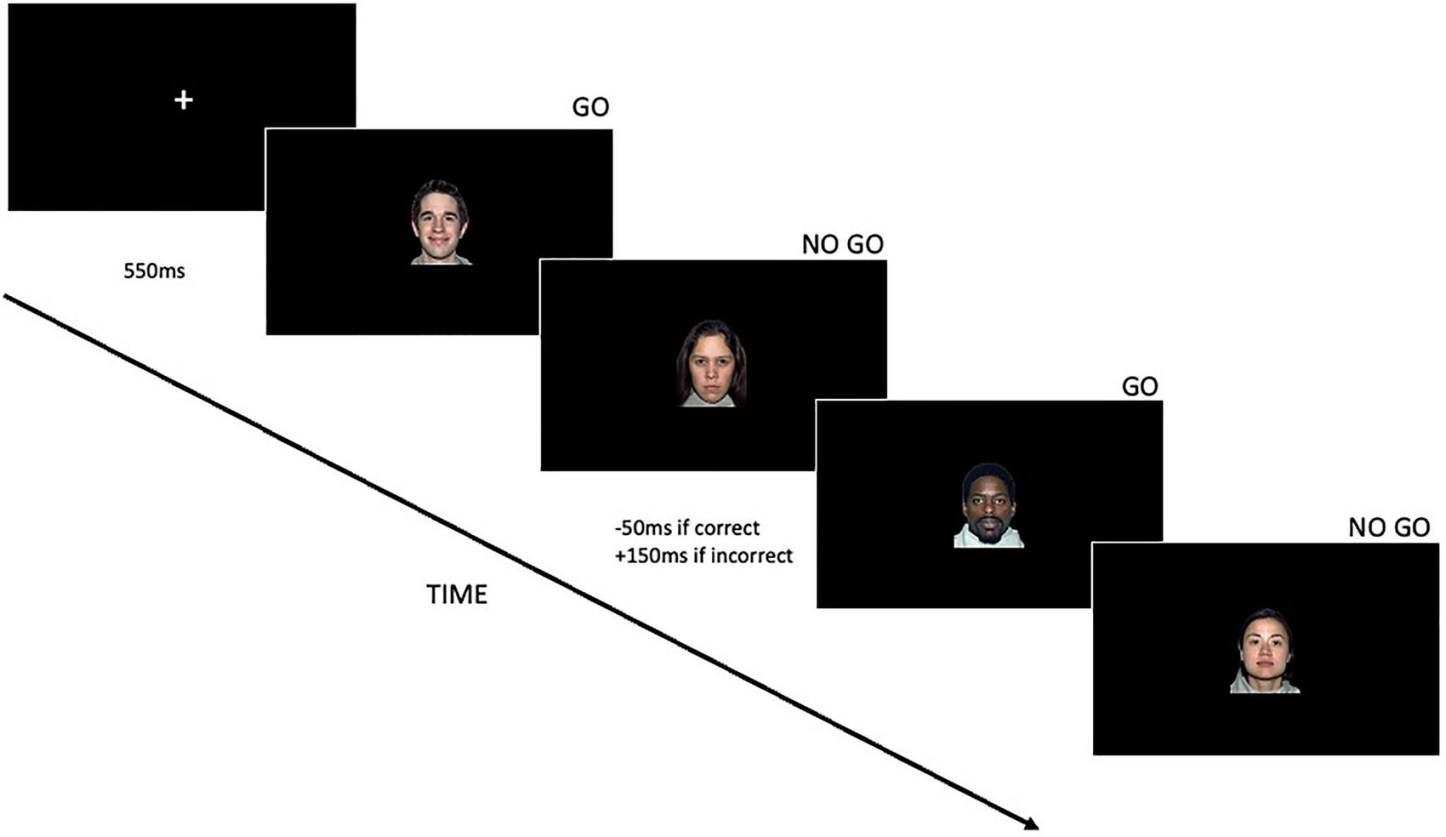
A visual representation of the emotional Go/No‐Go task used in the study.

An emotional version of the Go/No‐Go task was programmed and presented using E‐Prime Professional 2.0 software (Figure 1). The design of this task was a replication of the emotional Go/No‐Go task used by Hum, Manassis, and Lewis (2013a). Children were presented with four female models and four male models depicting angry, neutral, and happy emotions (closed mouth only) from the NimStim Face Stimulus Set on a screen (Tottenham et al. 2009). Models from different racial backgrounds were chosen to ensure children were presented with a diverse representation of faces. Children were asked to respond as fast and accurately as possible to a cue presented using a button box.

Gender was used as the Go/NoGo cue to capture automatic emotion regulation processes and was counterbalanced across participants. This implicit paradigm requires participants to execute a cognitive task that is unrelated to the emotional content, allowing us to explore emotion‐modulated cognitive processes (J. Zhang et al. 2023; W. Zhang et al. 2016; W. Zhang and Lu 2012). Importantly, research has shown that categorization of gender requires the face to be consciously perceived, whilst emotional facial expressions can be effectively processed in the absence of conscious awareness (Amihai, Deouell, and Bentin 2011). In our task, children were asked to respond when a male face appeared (Go trials) and not to respond to a female face (NoGo trials), or vice‐versa. Participants were given verbal instructions before the task started with visual instructions also included on the presentation screen. Checks of understanding were made by the experimenter before the task began.

Facial stimuli were edited to have a consistent black background and a size of 506 × 650 pixels. Following a white fixation cross‐appearing on the screen, all stimuli were presented in the center of the screen in a pseudo‐random order to ensure that the same identity of face did not follow each other. The task was dynamically adjusted based on the participant's performance to account for individual differences in skill level (see Supporting Information for minimum and maximum display durations). Go stimuli were initially displayed for 550 ms but this decreased by 50 ms following three correct responses or increased by 150 ms following three incorrect responses on NoGo trials (Hum, Manassis, and Lewis 2013a). The response window for NoGo trials was set to be 200 ms longer than Go trials to ensure that the nonresponse was deliberate. The task comprised of 20 practice trials followed by 2 blocks of 72 trials with a self‐controlled break. Each block consisted of 48 Go trials and 24 NoGo trials. Therefore, across the two blocks, each emotion was presented on 32 Go trials and 16 NoGo trials.

A total of four behavioral outcomes were obtained from the data: Go accuracy (%), NoGo accuracy (%), Go reaction time (RT), and NoGo RT. Accuracy rates and RTs were calculated for total trials and each emotion separately. The primary behavioral outcome measure of response inhibition performance was NoGo accuracy, which represents the proportion of responses successfully withheld. Lower levels of NoGo accuracy reflect poorer response inhibition performance. For overall behavioral data analysis, only responses made within 200 and 1200 ms of each trial were included to exclude nondeliberate responses (based on the procedure used by Hum, Manassis, and Lewis 2013a). Go accuracy, defined as the proportion of correct responses to Go stimuli, and RT on Go and NoGo trials, were reported to provide a detailed account of behavioral performance.

#### 2.3.2 Emotion Recognition Task

**FIGURE 2:**
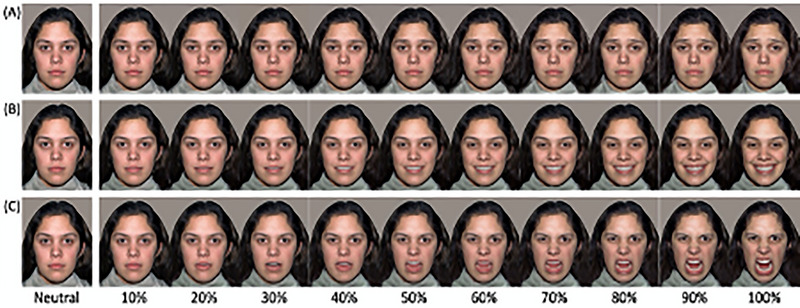
Stimuli used in the emotion recognition task. Children sorted photographs of sad (A), happy (B), and anger (C) expressions at increasing 10% intensity levels from neutral to sad, happy, or anger. Figure adapted from Birch‐Hurst et al. (2021). Copyright 2021 by Birch‐Hurst et al. Reprinted with permission from authors.

The stimuli were photographs of two female models selected from the NimStim Face Stimulus Set (Models 9 and 10; Tottenham et al. 2009) displaying expressions of happiness, sadness, and anger, as well as a neutral face. For each model, the three emotion expressions were morphed with the neutral face to create 10 levels of intensity, ranging 10%–100% (Figure 2; Gao and Maurer 2010). This resulted in 33 images for each model (3 expressions × 10 intensities + 3 neutral faces). Each image was printed in color (size: 9.5 × 12 cm), mounted onto a card and laminated.

This task was based on the procedure used by Gao and Maurer (2010) and Birch‐Hurst et al. (2021). Children were asked to place the cards, one‐by‐one, into one of four boxes corresponding to each emotion (happy, angry, sad, or neutral). In addition to verbal labels, each box was marked with a schematic face on the front and children were provided with prompts if they were struggling to choose a box (e.g., “try to go with your first thought” and “try not to think about it too much”).

The decision to include sadness in the emotion recognition task was to ensure that the participants were not just sorting on positive or negative valence but were also distinguishing between negative emotions. This is in line with the procedure used by Gao and Maurer (2010) and Birch‐Hurst et al. (2021), with the aim to have a more robust measure of emotion recognition.

We followed the scoring procedure used by Birch‐Hurst et al. (2021). Mean accuracy scores were calculated for each emotion, as well as an overall mean accuracy score. This was computed by averaging across each participants' accuracy for happy, angry, and sad expressions.

### 2.4 EEG Data Acquisition and Processing

The EEG was recorded from 32 channels using an electrode cap (ActiCap, Brain Products) with Ag/AgCl electrodes placed according to the International 10–20 system. An ActiCamp amplifier (Brain Products) was used and EEG activity was sampled at a rate of 500 Hz. The reference channel was Cz and ground was placed over Fpz. Impedances were kept below 30 kΩ, and channels were monitored during acquisition with noisy channels noted. Electrodes FT9, TP9, TP10, and FT10 were not included in the analysis due to poor signals across multiple participants.

The data were processed offline using MATLAB version R2021b (The MathWorks Inc. 2021). Data were initially bandpass filtered at 0.3–100 Hz and then re‐referenced to the average activity of all the electrodes. Artifacts in the data were automatically identified using a threshold value of ±200 µV, and then excluded from the data. Eye blinks were automatically identified as signals that met predefined thresholds of > 100 ms rise time, > 150 ms fall time, and > 125 µV amplitude at electrodes Fp1 and Fp2, and visual inspection follow‐up to ensure appropriate exclusion of blinks. Practice trials and those with anticipatory responses (RTs < 200 ms) were also removed from the data. The data were then low‐pass filtered at 30 Hz for ERP construction. Cleaned data were then segmented. Stimulus‐locked ERPs (N2, P3) for Go and NoGo trials were segmented into 100 ms pre‐stimulus baseline to 1000 ms post‐stimulus epochs. ERPs were further split according to trial type (Go and NoGo stimuli) for each of the three different emotions in the task (happy, angry, and neutral).

The N2 was scored from 300 to 400 ms and P3 scored from 480 to 600 ms in Fz. In addition to separate stimulus‐locked Go‐ and NoGo–ERPs, difference waveforms were computed for P3 and N2 (“P3d,” “N2d”), with NoGo amplitudes minus Go amplitudes (e.g., P3d = P3_NoGo_ − P3_Go_ amplitudes). This procedure is used to isolate the unique effects of NoGo–ERPs by controlling for effects common across both Go and NoGo trials (Bekker, Kenemans, and Verbaten 2005; Gajewski and Falkenstein 2013).

These time windows were established through comparison with previous literature within the same age range (Davies, Segalowitz, and Gavin 2004; Hum, Manassis, and Lewis 2013a; Santesso, Segalowitz, and Schmidt 2006; Taylor et al. 2001) and visual inspection of individual participant and grand mean plots. Mean amplitudes were used for most statistical analyses as this is reported to be a more robust measure of ERP waveforms than peak amplitude (Clayson, Baldwin, and Larson 2013). The average number of valid trials used in ERP analyses for Go (out of 32 trials per emotion) and NoGo conditions (out of 16 trials per emotion) were similar across emotions (Angry Go: *M* = 18.27, SD = 6.26, 4–30; Angry NoGo: *M* = 9.92, SD = 2.44, 4–14; Neutral Go: *M* = 18.52, SD = 6.57, 3–31; Neutral NoGo: *M* = 10.10, SD = 2.78, 3–15; Happy Go: *M* = 18.37, SD = 6.95, 3–31; Happy NoGo: *M* = 10.13, SD = 3.22, 3–16).

### 2.5 Procedure

First, the researcher took head circumference measurements and began capping the child while they completed self‐report questionnaires. Children who were recruited in Stage 1 completed the ChEAT and RCADS‐25 in their schools within small groups (as described in Thomas et al. 2021) and did not have to repeat the questionnaires. Once the EEG cap had been fitted and electrode gel applied, the child was sat in a separate testing room to complete the tasks.

The EEG session began with a resting session, where baseline EEG data were collected with six 30s blocks in which participants were instructed to alternate between keeping their eyes opened or closed for the 30‐s duration. Overall, the tasks lasted approximately 10 min. In addition to this emotional Go/No‐Go task, the child completed a nonemotional version of the Go/No‐Go task as part of a larger project. The order of these two tasks was counterbalanced across participants (emotional Go/No‐Go task first, *n* = 26, non‐emotional Go/No‐Go task first, *n* = 27).

### 2.6 Statistical Analyses

All statistical analyses were conducted using SPSS (version 27.0; IBM 2020). RCADS‐25 data violated the assumption of normality based on visual inspection of histograms and the Shapiro–Wilk test of normality. This was corrected using a Log10(+1) transformation. *t* Tests revealed no significant differences in the accuracy and RT data collected for Go and NoGo trials on the Go/No‐Go task when compared across the two counterbalance groups. This was also the case when we checked for order effects of the two Go/No‐Go tasks (further details provided in Tables S1 and S2). Preliminary analyses investigated gender and age effects on behavioral and ERP data. Gender was added as a covariate in both Go/NoGo and emotion recognition behavioral analyses as there were significant differences found between boys and girls for accuracy on NoGo trials (*M* accuracy boys = 68.90, SD = 7.55; *M* accuracy girls = 73.47, SD = 8.65; *t*(51) = −2.05, *p *= 0.045) and recognition accuracy for sad emotional expressions (*M* accuracy boys = 64.17, SD = 12.18; *M* accuracy girls = 73.70, SD = 8.42; *t*(51) = −3.21, *p* = 0.002). Age was added as a covariate in analyses involving stimulus‐locked ERPs across all emotion conditions, as age was significantly associated with N2 and P3 amplitudes on Neutral Go trials (N2: *r* = 0.386, *p* = 0.005; P3: *r* = −0.320, *p* = 0.021). Finally, we investigated potential interaction effects between the child's gender and the gender of the actors in the Go/NoGo stimuli. A *χ* ^2^ test for association was conducted between gender of the child and the Go/NoGo counterbalance condition. All expected cell frequencies were greater than 5. There was not a statistically significant association between gender and counterbalance condition, *χ* ^2^(1) = 0.046, *p* = 0.829.

Unless specified, untransformed data are presented in tables. Homogeneity of variance was assessed by Levene's test for equality of variances. Where this was violated, comparative non‐parametric tests were used, for example, Mann–Whitney *U*. Bonferroni correction was used to adjust for multiple comparisons.

To test our primary hypotheses and investigate the associations between DE, internalizing symptoms, and measures of emotion regulation, Pearson's *r* correlations were conducted. Where significant correlations were present between DE and emotion regulation (for either behavioral or ERP measures), follow‐up hierarchical regression analyses were conducted to examine the association between DE and emotion regulation, while controlling for anxiety and depression. For regression analyses, multicollinearity was tested using the variance inflation factor and at an acceptable level (Neter, Wasserman, and Kutner 1985), unless reported otherwise. Finally, to establish whether emotion recognition was associated with DE and internalizing symptoms, Pearson's correlations were conducted.

To examine the relation between DE and stimulus‐locked ERPs (P3, N2) across different emotions, difference waveforms (N2d, P3d) were calculated and used in place of individual ERPs. This is in line with previous emotional Go/NoGo tasks used with adolescents to capture the N2_NoGo_ and P3_NoGo_ effects more clearly across different emotions (Sun et al. 2020). Finally, we computed difference waveforms to isolate the effects of emotional content from the early negative deflection commonly linked to face processing (emotional vs. neutral contexts).

## 3 Results

The majority of participants reported low levels of DE, anxiety, and depressive symptoms (Table 1), aligning with mean scores reported in other community samples of children and adolescents using these measures (Anton et al. 2006; Carlander et al. 2024; Thomas et al. 2021). Performance on the Go/NoGo task was acceptable (Table 2). ChEAT scores were positively correlated with both RCADS Anxiety (*r* = 0.64, *p* < 0.001) and RCADS Depression scores (*r* = 0.43, *p* = 0.001). Analyses revealed no significant correlations between Go/NoGo task performance and ChEAT scores, RCADS anxiety, and RCADS depression.

**TABLE 2:** Go/NoGo behavioral data for the whole sample (*N* = 53).

	Emotion			
	Overall	Angry	Neutral	Happy
Go accuracy (%)	72.58 (19.18)	71.29 (19.39)	72.88 (18.90)	71.82 (20.30)
NoGo accuracy (%)	70.89 (8.28)	72.10 (12.92)	69.29 (14.55)	68.75 (14.88)
Go mean RT (ms)	440.70 (59.91)	443.30 (62.74)	438.07 (62.09)	440.68 (64.38)
NoGo mean RT (ms)	372.95 (67.46)	377.16 (76.65)	366.80 (88.48)	375.00 (74.75)

*Note:* Values reflect means with standard deviation in parentheses.

Participants were generally consistent in their emotion recognition performance across each emotional expression; however, accuracy was lower for happy and sad expressions compared to anger (Table 3). Higher ChEAT scores were associated with lower mean recognition accuracy on happy trials (*r* = −0.34, *p* = 0.01) and this correlation remained significant when Bonferroni correction was used to adjust for multiple comparisons (corrected *α* level = 0.02).

**TABLE 3:** Accuracy (%) of each labelled emotional expression and correlations between questionnaire and emotion recognition performance for each emotion.

	*M* (SD)	ChEAT	RCADS anxiety	RCADS depression
Happy	67.26 (8.00)	−0.34^*^	−0.22	−0.09
Anger	75.75 (7.68)	−0.05	−0.06	0.08
Sad	68.30 (11.64)	−0.04	0.04	−0.05

*Note:* Accuracy (%) is of correctly labelled photographs out of 20.

Abbreviations: ChEAT, Children's Eating Attitude Test; RCADS, Revised Child Anxiety Depression Scale.

* *p* < 0.05.

### 3.1 Stimulus‐Locked ERPs

**FIGURE 3:**
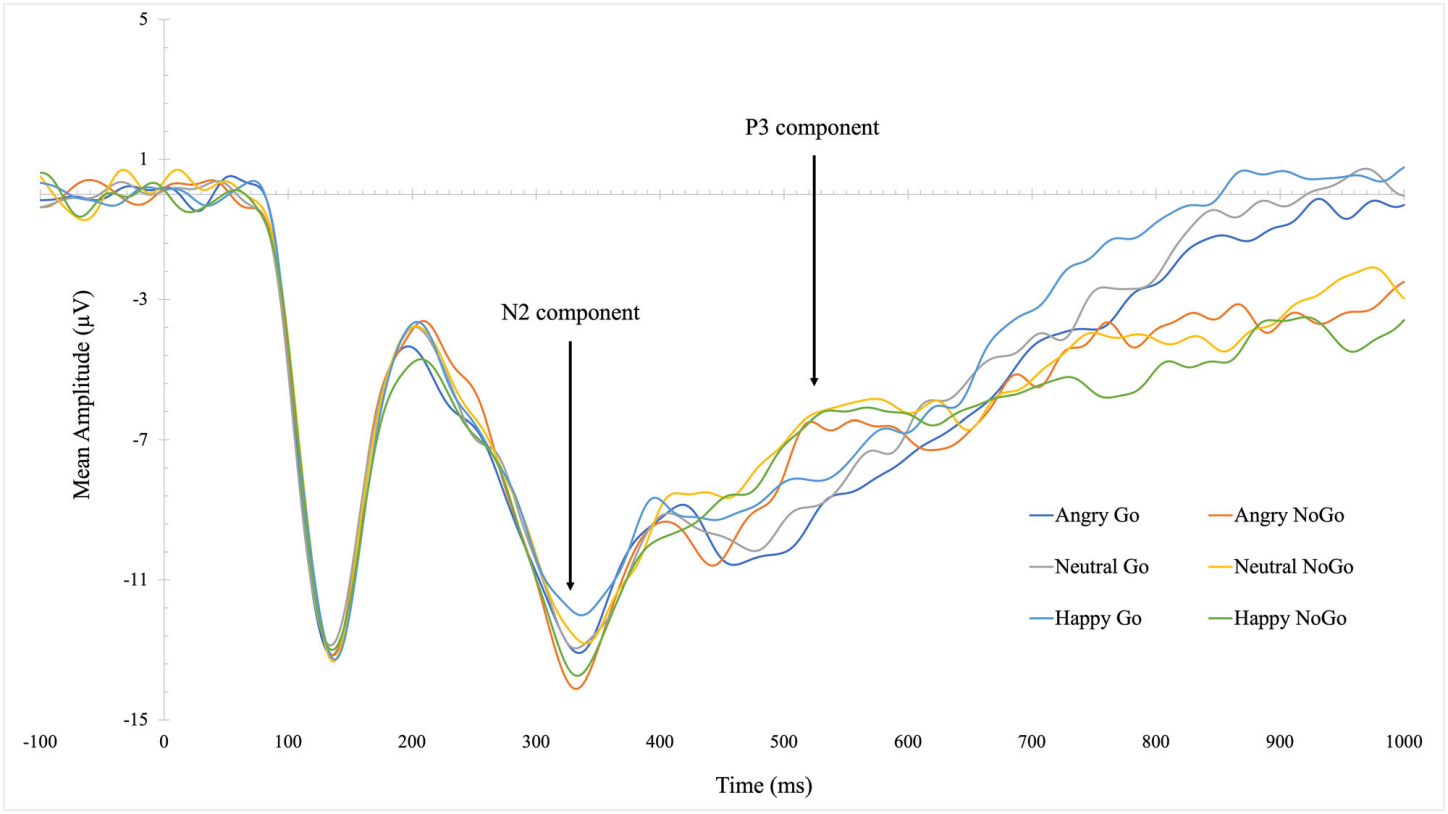
Grand mean stimulus‐locked waveforms for each emotion (anger, neutral, and happy) on Go and NoGo trials at Fz (*N* = 52).

Figure 3 presents grand mean stimulus‐locked waveforms for each emotion (anger, neutral, and happy) on Go and NoGo trials. As expected, both the N2 and P3 components were larger for NoGo trials, compared to Go trials, across most emotions. The exception was N2 amplitudes on neutral trials, which were similar across Go and NoGo trials.

#### 3.1.1 Primary Analyses

To test our hypothesis, we examined whether DE and internalizing symptoms would be correlated with attenuated P3 amplitudes and enhanced N2 amplitudes on NoGo trials. First, we isolated the effect of NoGo trials from Go trials across each emotion by calculating a difference score for P3 and N2 (e.g., P3d = P3_NoGo_ − P3_Go_ amplitudes; N2d = N2_NoGo_ − N2_Go_ amplitudes). We found a significant positive correlation between P3d amplitudes for happy trials and ChEAT scores (*r* = 0.32, *p* = 0.02). This demonstrates an enhancement in the P3_NoGo_ effect with happy faces as DE increases. There was also a significant negative correlation between P3d amplitudes on neutral trials and anxiety (*r* = −0.41, *p* = 0.01) and depression (*r* = −0.32, *p* = 0.02). However, the correlation with DE was not significant (*r* = −0.26, *p* = 0.07). This suggests a decreasing P3_NoGo_ amplitude with neutral faces as anxiety and depression symptoms increase.

The significant correlation between ChEAT scores and P3d amplitudes on happy trials was followed by a hierarchical multiple regression to control for the effects of internalizing symptoms (Table 4). Anxiety and depression were added to the model at Step 1, and P3d amplitudes were added at Step 2. The full model of P3d_happy_ amplitudes, anxiety, and depression in relation to ChEAT was statistically significant; however, anxiety was the only significant coefficient in Steps 1 and 2. In addition, the *F*‐value did not significantly change between steps, suggesting P3d_happy_ amplitudes were not able to account for significant variability in ChEAT scores over and above internalizing symptoms alone. Difference waveforms calculated for N2 amplitudes were not associated with DE or internalizing symptoms, so were not explored any further.

**TABLE 4:** Hierarchical multiple regression of P3d amplitudes on Happy trials and anxiety, and depression on Children's Eating Attitude Test (ChEAT) scores.

Variable	*B*	95% CI for *B*		SE *B*	*β*	*t*	*p*
		LL	UL				
Step 1							
Constant	29.20	18.29	40.11	5.43		5.38	< 0.001
Anxiety	28.79	14.99	42.58	6.86	0.63	4.19	< 0.001
Depression	0.38	−12.99	13.75	6.65	0.01	0.06	0.96
*R* ^2^	0.40						
*F*	16.24						< 0.001
Step 2							
Constant	30.73	19.78	41.68	5.45		5.64	< 0.001
Anxiety	26.24	12.21	40.26	6.98	0.57	3.76	< 0.001
Depression	1.21	−12.03	14.46	6.59	0.03	0.18	0.86
P3d_happy_	0.19	−0.06	0.44	0.12	0.17	1.53	0.13
*R* ^2^	0.43						
*F*	11.89						< 0.001
Δ*R* ^2^	0.03						
Δ*F*	2.33						0.13

*Note:* P3d: P3_NoGo_ − P3_Go_ difference wave for happy trials. Steps were defined in the same hierarchical regression analysis. Transformed data were used in the analyses.

Abbreviations: *B*, unstandardized regression coefficient; CI, confidence interval; LL, lower limit; SE *B*, standard error of the coefficient; UL, upper limit; *β*, standardized coefficient; *R* ^2^, coefficient of determination; Δ*R* ^2^, *R* ^2^ change; Δ*F*, *F*‐value change.

In an additional step to our analysis, we calculated difference scores to account for neural activity linked to face processing in our stimulus‐locked ERP amplitudes. These difference scores were calculated for Go and NoGo trials by subtracting the amplitude of neutral emotion trials from emotion (Angry and Happy) trials (e.g., P3d_Go_ Angry = P3_Go_ Angry − P3_Go_ Neutral; N2d_NoGo_ Happy = N2_NoGo_ Happy − N2_NoGo_ Neutral). As we did with the difference scores above, we then isolated the effect of the NoGo trials, resulting in four difference scores: N2_NoGo_ and P3_NoGo_ difference waveforms for both Angry and Happy emotions. When we repeated the correlational and regression analyses reported for the original difference scores using these difference waveforms, we found consistent findings (full results are provided in Tables S3 and S4), suggesting these effects were not driven by neural activity linked to face processing.

## 4 Discussion

This study explored the link between DE, internalizing symptoms, and both neural and behavioral correlates of response inhibition in a community sample of preadolescents. The key finding from this study is the relation between increased DE and elevated inhibitory effort for happy faces at a neural level in preadolescence. This association was not found to be present with internalizing symptoms, suggesting that although there is a strong association between DE and internalizing symptoms in preadolescence, these response inhibition difficulties appear to be associated with DE only.

Our neural findings suggest preadolescents with higher levels of DE display response inhibition difficulties in the context of happy faces, specifically for the P3 amplitude. We calculated difference waves to isolate the unique effects of P3_NoGo_, the neural marker of inhibitory effort, by controlling for effects common across both Go and NoGo trials (Bekker, Kenemans, and Verbaten 2005; Gajewski and Falkenstein 2013). Individuals with higher levels of DE displayed enhanced P3d amplitudes for happy faces, but similar amplitudes to those with lower levels of DE for angry and neutral faces. Moreover, this effect was independent of internalizing symptoms. This is inconsistent with our hypothesis that increased levels of DE and internalizing symptoms would be positively correlated with neural markers of impaired response inhibition (in the form of less positive P3 amplitudes), based on previous reports of attenuated P3 amplitudes during emotional processing in individuals with anorexia nervosa (Hatch et al. 2010; Pollatos et al. 2008). However, when compared to the processing of happy expressions specifically, our findings are consistent with fMRI research, which reports individuals with anorexia nervosa to display greater neural activity to increasing intensity of happy expressions compared to healthy controls (Fonville et al. 2014). The authors propose this to be reflective of the increased salience of positive expressions (Fonville et al. 2014). Indeed, increased startle responses have been found in individuals with anorexia nervosa when viewing positive stimuli, as well as body and food stimuli (Friederich et al. 2006). Enhanced P3 amplitudes have also been reported during the presentation of food stimuli compared to neutral stimuli in adolescents with higher levels of loss of control eating (Biehl et al. 2019) and emotional eating (J. Wu et al. 2018). Attenuated P3 amplitudes are instead found during neutral tasks in individuals with anorexia nervosa (Bradley et al. 1997; Yue et al. 2020) and preadolescents with higher levels of DE (Thomas et al. 2024). Combined with the current findings, these results suggest that higher levels of DE are associated with enhanced P3 amplitudes (i.e., greater inhibitory effort) when processing emotive stimuli, such as positive face expressions and food stimuli. As the anterior cingulate cortex (ACC) is the neural generator of the P3 (L. Zhang et al. 2012), our findings also have implications for its function. For example, elevated ACC activity in response to food stimuli has been proposed to be a trait marker for anorexia nervosa (Frank et al. 2004; Uher et al. 2003). Overall, these findings suggest hyperactivity of the ACC in response to emotive stimuli may be an early indicator of increased ED risk.

Although not the focus of our study, we found increased DE to also be associated with poorer recognition of happy facial expressions, providing support for a specific difficulty in happy face processing in children with high levels of DE. Previous research has identified emotion recognition deficits in individuals with EDs (Harrison et al. 2009; Harrison, Tchanturia, and Treasure 2010) and those with high levels of DE (Ridout, Thom, and Wallis 2010), but these studies report a global emotion deficit, rather than one specific to happy faces. However, our findings are more consistent with evidence in individuals with anorexia nervosa showing a reduced capacity to process positive emotion expressions compared to healthy controls and individuals with obesity (Cserjési et al. 2011). This is the first study to examine emotion recognition performance in preadolescents with DE behaviors. The emotion recognition measure used in our study varied the intensity of expression by morphing from neutral to full expression. While previous studies have used static images of full expressions (Harrison et al. 2009; Harrison, Tchanturia, and Treasure 2010; Sharpe et al. 2016), using more complex stimuli may have given us the ability to identify a more nuanced relation between DE and emotion recognition.

When discussing our findings, it is also important to highlight some of the limitations of our research. Firstly, the emotion recognition task included female models only. However, comparisons between counterbalancing orders showed no differences when males or females were the Go or the NoGo cue. Another limitation is the issue of task impurity, a commonly reported measurement problem in the executive functioning literature, which proposes that performance on a task reflects variation in a number of cognitive processes, rather than just measuring the function of interest (Best and Miller 2010; Hughes and Graham 2002; Miyake et al. 2000). Across previous studies, a variation of tasks are used across age ranges, making comparisons across development challenging (Klenberg et al. 2015). To try and mitigate these issues in our study, the demands of the Go/NoGo task used were dynamically adjusted based on the child's performance. This meant children should have experienced a similar level of difficulty, regardless of their underlying proficiency on the task. Finally, it is important to acknowledge the cross‐sectional nature of the research. Currently, we are unable to comment on any causal relations; so replication and extension of this research is required. For example, it may be the case that DE is leading to enhanced P3 amplitudes. Longitudinal designs would enable trajectories of DE and internalizing symptoms across development to be examined, providing more insight into the etiology of DE. This investigation would be particularly important given the onset of potential life stressors during this developmental stage (e.g., transition to secondary school and puberty), which can increase the risk for mental health difficulties (Low et al. 2012; Riglin et al. 2013). Therefore, collecting data across multiple time points, from preadolescence (age 10–11 years) to early adolescence (12–13 years), would allow us to probe these trajectories of DE across a life stressor, such as the transition from primary to secondary school.

Future research should explore a wider range of emotions in the context of DE. For example, disgust has been frequently implicated in the development and maintenance of EDs (Fox and Froom 2009; Fox and Harrison 2008; Fox and Power 2009; Harvey et al. 2002; Troop, Treasure, and Serpell 2002). Disgust can function as a threat‐related emotion, potentially contributing to ED‐related avoidance behaviors, such as food avoidance and calorie restriction (Anderson et al. 2021). Positive emotions that go beyond happiness, such as pride, should also be explored, along with the integration of information from the wider context, such as body cues. For example, emotional expressions that map onto positive and rewarding events, such as winning a game or completing a task, may provide us with more information about the processing of positive emotional stimuli in EDs. Additional co‐occurring factors should also be considered in research examining at‐risk samples, such as the effects of autistic traits and alexithymia, as both have previously been shown to be associated with emotion recognition difficulties in EDs (Brewer et al. 2015; Kerr‐Gaffney et al. 2020).

In conclusion, the findings from this study provide a novel contribution to our understanding of response inhibition within an emotional context and DE in preadolescents. Results suggest a relation between increased DE and impaired happy face processing at a neural level in preadolescence. An early disruption in response inhibition may be relevant to the development of DE behaviors and the potential for developing diagnosable EDs.

## Ethics Statement

Ethical approval was received from the Cardiff University School of Psychology Ethics Committee before research commenced. Opt‐in parent/guardian consent and child assent were obtained, and all data were stored anonymously. The Research was conducted as per British Psychological Society guidelines and the Economic and Social Research Council Research Ethics Framework.

## Conflicts of Interest

The authors declare no conflicts of interest.

## Supporting information



Table S1. Differences in Go/No‐Go accuracy and reaction time (RT) data across counterbalance groups.
Table S2. Differences in Go/No‐Go accuracy and reaction time (RT) data across order of Go/No‐Go tasks.
Table S3. Minimum and maximum stimuli durations across conditions.
Table S4. Hierarchical multiple regression of P3_NoGo_ Happy difference scores, anxiety, depression, and recruitment type on children's eating attitude test (ChEAT) scores.

## Data Availability

Data is available at https://osf.io/h3b24/.
