# Supplementary material for: Neural Correlates of Emotion Regulation and Associations With Disordered Eating During Preadolescence
Source: Dev Psychobiol. 2024 Dec 8;67(1):e70009. doi: 10.1002/dev.70009 (PMC11625878; doi:10.1002/dev.70009)
Supplement: Supplementary file 1 — Table S1. Differences in Go/No‐Go accuracy and reaction time (RT) data across counterbalance groups. Table S2. Differences in Go/No‐Go accuracy and reaction time (RT) data across order of Go/No‐Go tasks. Table S3. Minimum and maximum stimuli durations across conditions. Table S4. Hierarchical multiple regression of P3NoGo Happy difference scores, anxiety, depression, and recruitment type on children's eating attitude test (ChEAT) scores. [file DEV-67-e70009-s001.docx]

Supplementary Materials

**Table S1.**

*Differences in Go/No-Go accuracy and reaction time (RT) data across counterbalance groups.*

|  | Group 1 | Group 2 | *t*(51) | *p* |
| --- | --- | --- | --- | --- |
|  | *M* (SD) | *M* (SD) |  |  |
| Go Accuracy (%) | 76.00 (19.58) | 69.54 (18.64) | 1.23 | .22 |
| NoGo Accuracy (%) | 70.43 (7.30) | 71.29 (9.19) | -.38 | .71 |
| Go RT (ms) | 439.09 (64.15) | 442.13 (57.00) | -.18 | .86 |
| NoGo RT (ms) | 357.99 (56.39) | 386.31 (74.46) | -1.55 | .128 |

*Note.* Counterbalance group 1 (n = 25) = Female Go target, Counterbalance group 2 (n = 28) = Male Go target.

**Table S2.**

*Differences in Go/No-Go accuracy and reaction time (RT) data across order of Go/No-Go tasks*

|  | Order 1 | Order 2 | *t*(51) | *p* |
| --- | --- | --- | --- | --- |
|  | *M* (SD) | *M* (SD) |  |  |
| Go Accuracy (%) | 68.67 (18.56) | 76.65 (19.33) | -1.54 | .13 |
| NoGo Accuracy (%) | 71.87 (7.99) | 69.87 (8.62) | .88 | .38 |
| Go RT (ms) | 445.03 (60.77) | 436.20 (59.86) | .53 | .60 |
| NoGo RT (ms) | 385.81 (70.95) | 359.60 (62.18) | 1.43 | .16 |

*Note.* Order 1 (n = 27) = Non-emotional task first, Order 2 (n = 26) = Emotional task first.

**Table S3.**

*Minimum and maximum stimuli durations across conditions.*

|  |  | Sample range (ms) | Sample average (ms) |
| --- | --- | --- | --- |
| All trials |  |  |  |
|  | Minimum | 150 – 900 | 334.91 |
|  | Maximum | 800 - 1600 | 1273.58 |
| Go trials |  |  |  |
|  | Minimum | 150 – 900 | 334.91 |
|  | Maximum | 600 - 1400 | 1074.53 |
| NoGo trials | |  |  |
|  | Minimum | 350 – 1100 | 533.02 |
|  | Maximum | 800 - 1600 | 1273.58 |
| Female model trials | |  |  |
|  | Minimum | 150 - 1100 | 439.62 |
|  | Maximum | 650 - 1600 | 1180.19 |
| Male model trials | |  |  |
|  | Minimum | 150 - 1050 | 428.30 |
|  | Maximum | 600 - 1600 | 1180.19 |
| Angry trials | |  |  |
|  | Minimum | 150 - 950 | 353.77 |
|  | Maximum | 750 - 1600 | 1226.42 |
| Happy trials | |  |  |
|  | Minimum | 150 - 900 | 348.11 |
|  | Maximum | 650 - 1600 | 1228.30 |
| Calm trials | |  |  |
|  | Minimum | 150 – 950 | 342.45 |
|  | Maximum | 650 - 1600 | 1228.30 |

Difference waveforms isolating the effects of emotion content

We calculated difference waveforms to isolate the emotion content from the early negative reflection in neural activity linked to face processing in an additional stage to our analysis. We found a significant positive correlation between ChEAT scores and P3_NoGo_ Happy difference scores (*r* = .36, *p* = .01), but the correlation between ChEAT scores and P3_NoGo_ Angry difference scores was not significant (*r* = .26, *p* = .06). For anxiety and depressive symptoms, only anxiety was found to be significantly correlated with P3_NoGo_ Happy difference scores (*r* = .38, *p* = .01) and P3_NoGo_ Angry difference scores (*r* = .34, *p* = .01). These findings suggest an enhancement in the P3_NoGo_ effect for emotional faces as anxiety symptoms and DE increase.

When we conducted these analyses separately for each recruitment type (pre- vs post-pandemic), we found some meaningful differences in the reported correlations between groups. Firstly, in the pre-pandemic group we found a significant positive correlation between P3_NoGo_ Angry difference scores and anxiety symptoms (*r* = .47, *p* = .04), but this was not significant in the post-pandemic group (*r* = .25, *p* = .16). In addition, a significant positive correlation was found between anxiety and P3_NoGo_ Happy difference scores for the post-pandemic group (*r* = .35, *p* = .04), but this was not significant in the pre-pandemic group (*r* = .39, *p* = .10). Therefore, we controlled for recruitment type in our subsequent regression analyses.

These significant correlations between ChEAT scores and P3_NoGo_ Happy difference scores were followed by hierarchical multiple regressions to control for the effects of internalizing symptoms (Table S4). Anxiety, depression, and recruitment type were added at Step 1 of the model and P3_NoGo_ Happy difference scores were added at Step 2. As displayed in Table S3, the full model of P3_NoGo_ Happy difference scores, anxiety, depression, and recruitment type in relation to ChEAT was statistically significant; however, anxiety was the only significant coefficient at Step 1 and 2. In addition, the *F*-value did not significantly change between steps, suggesting P3_NoGo_ Happy difference scores were not able to account for significant variability in ChEAT scores over and above internalizing symptoms alone.

**Table S4.***Hierarchical multiple regression of P3_NoGo_ Happy difference scores, anxiety, depression, and recruitment type on children’s eating attitude test (ChEAT) scores.*

| Variable | *B* | 95% CI for B | | *SE B* | 𝛃 | *t* | *p* |
| --- | --- | --- | --- | --- | --- | --- | --- |
|  |  | *LL* | *UL* |  |  |  |  |
| Step 1 |  |  |  |  |  |  |  |
| Constant | 36.29 | 18.02 | 54.56 | 9.09 |  | 3.99 | <.001 |
| Anxiety | 28.61 | 14.80 | 42.42 | 6.87 | .62 | 4.16 | <.001 |
| Depression | -1.68 | -15.72 | 12.37 | 6.99 | -.04 | -.24 | .81 |
| Recruitment type | -3.17 | -9.71 | 3.38 | 3.26 | -.12 | -.97 | .34 |
| *R^2^* | .41 |  |  |  |  |  |  |
| *F* | 11.13 |  |  |  |  |  | <.001 |
| Step 2 |  |  |  |  |  |  |  |
| Constant | 37.624 | 19.263 | 55.985 | 9.127 |  | 4.122 | <.001 |
| Anxiety | 26.012 | 11.529 | 40.496 | 7.199 | .565 | 3.613 | .01 |
| Depression | -1.369 | -15.381 | 12.643 | 6.965 | -.031 | -.196 | .85 |
| Recruitment type | -3.077 | -9.606 | 3.451 | 3.245 | -.115 | -.948 | .35 |
| P3_NoGo_ Happy | .115 | -.083 | .313 | .098 | .139 | 1.164 | .25 |
| *R^2^* | .43 |  |  |  |  |  |  |
| *F* | 8.75 |  |  |  |  |  | <.001 |
| 𝜟*R^2^* | .02 |  |  |  |  |  |  |
| 𝜟*F* | 1.36 |  |  |  |  |  | .25 |

*Notes*. P3_NoGo_ Happy: P3_NoGo_ Happy difference wave. Recruitment type: 1 = pre-pandemic, 2 = post-pandemic. Steps were defined in the same hierarchical regression analysis; *B* = unstandardized regression coefficient; CI = confidence interval; *LL* = lower limit; *UL* = upper limit; *SE B* = standard error of the coefficient; 𝛃 = standardized coefficient; *R*^2^ = coefficient of determination; 𝜟*R^2^* = *R* square change; 𝜟*F* = *F* value change. Transformed data were used in the analyses.
